# Protective Activities of Growth Hormone-Releasing Hormone Antagonists against Toxin-Induced Endothelial Injury

**DOI:** 10.3390/endocrines5010008

**Published:** 2024-03-18

**Authors:** Saikat Fakir, Nektarios Barabutis

**Affiliations:** School of Basic Pharmaceutical and Toxicological Sciences, College of Pharmacy, University of Louisiana Monroe, Monroe, LA 71201, USA

**Keywords:** inflammation, growth hormone, kinases, growth factors

## Abstract

GHRH regulates the secretion of GH from the anterior pituitary gland, previously associated with cancer progression and inflammation. An emerging body of evidence suggests that GHRHAnt support endothelial barrier function, but the mechanisms mediating these events are not completely understood. In the present study, it is demonstrated that the GHRHAnt JV-1-36 counteracts barrier dysfunction due to LPS or LTA treatment in HUVECs, utilizing the Dextran-FITC assay. Moreover, it is shown in BPAECs that these bacterial toxins increase ROS generation, and that this effect is counteracted by JV-1-36, which reinstates the redox balance. The possible involvement of NEK2 in the beneficial activities of GHRHAnt in IFN-γ- and LPS-triggered hyperpermeability was also assessed, since that kinase is involved in inflammatory responses. NEK2 was increased in the inflamed cells, and JV-1-36 counteracted those endothelial events. Our data support the beneficial effects of GHRHAnt in toxin-induced endothelial injury.

## 1. Introduction

Endothelial barrier dysfunction has been associated with a diverse variety of potentially lethal disorders, including direct and indirect lung injury, acute respiratory syndrome, and sepsis. Targeted efficient countermeasures for the aforementioned disorders are urgently needed, as indicated by the devastating outcomes of COVID-19 [1]. The delineation of the cellular pathways mediating endothelial homeostasis will most probably lead to the development of novel pharmacotherapies to support the inflamed endothelium and respiratory function.

In the present study, endothelial cells were treated with LPS, LTA, and IFN-γ. LPS exists in Gram-negative bacteria, whereas LTA is the endotoxin of Gram-positive bacteria and induces proinflammatory responses. IFN-γ is a downstream mediator of both bacterial toxins. Both human (HUVECs) and bovine endothelial cell lines (BPAECs) were cultured to study barrier function. Dextran–FITC was used to measure the paracellular permeability in the context of GHRHAnt in toxin-induced barrier dysfunction.

GHRHAnt are peptides designed to alleviate the inflammatory and tumor-promoting effects of the GH/GHRH axis in human and animal tissues [2,3] and act—at least in part—by binding to the growth hormone-releasing hormone receptor and its splice variants. Those receptors are expressed in various tissues and endothelial cells [4–8], including BPAECs [9]. GHRHAnt were initially synthesized to suppress tumors in experimental models of malignancies. Later on, it was recognized that they can exert beneficial effects in inflammatory lung disease, based on their ability to suppress ERK1/2 and JAk2/STAT3; and augment the wild-type P53. This tumor suppressor enhances barrier function and protects against inflammation. P53 deficient mice are more susceptible to LPS compared to animals expressing normal P53 levels, and super-P53 mice were resilient to LPS-induced lung injury [10]. P53 interrelates with NEK2, since it serves as an NEK2 substrate.

NEK2 is a kinase involved in cytoskeletal remodeling, cell motility, and cell cycle regulation, participating in microtubule formation, stabilization, and centrosome regulation [11–13]. Mice subjected to cecal and ligation-puncture-induced sepsis presented with elevated NEK2 expression levels in their lungs [14], and studies on experimental cancers suggest that this kinase is involved in cancer progression and drug resistance [15]. Indeed, NEK2 inhibition was suggested to be a potential anticancer strategy [16–18].

In the present study, we investigated the possibility that GHRHAnt protect against toxin-induced endothelial barrier dysfunction in the context of paracellular permeability and ROS generation. Our observations suggest that GHRHAnt ameliorate endothelial leak and oxidative stress due to bacterial toxin treatment [19]. Furthermore, NEK2 was induced in the inflamed cells, and GHRHAnt counteracted those effects. Since NEK2 was previously linked to inflammatory responses, we introduce the possibility that this kinase is involved in the GHRHAnt-related beneficial effects in the endothelium [20,21].

## 2. Materials and Methods

### 2.1. Reagents

GHRHAnt JV-1-36 (031-23) is available from Phoenix Pharmaceuticals (Burlingame, CA, USA). Anti-mouse IgG HRP secondary antibody (95017-554), nitrocellulose membranes (10063-173) (BT142015-5G), IFN-γ (103014-494), and RIPA solution (AAJ63306-AP) were purchased from VWR (Radnor, PA, USA). Protease Inhibitor (AB287909) was purchased from Abcam (Cambridge, UK). NEK 2 (D-8) (sc-55601) antibody is available from Santa Cruz Biotechnology (Dallas, TX, USA). β-actin antibody (A6441), Corning trans-well cell culture inserts (CLS3470), LPS (L4130), and FITC–Dextran (46945) were acquired from Sigma-Aldrich (St. Louis, MO, USA).

### 2.2. Cell Cultures

Bovine pulmonary artery endothelial cells (BPAECs) are available from Genlantis (San Diego, CA, USA). The cells were maintained in DMEM (VWRL0101–0500), which included 10% fetal bovine serum, available from VWR (Radnor, PA, USA). Human umbilical vein endothelial cells (HUVECs) were subcultured in media specific to endothelial cells (PCS-100–030), and a special kit was purchased (PCS-100–040) to supplement it. The material is available from ATCC (Manassas, VA, USA). Moreover, to prevent infections/contaminations, we added a solution of 1 × penicillin/streptomycin to the cell media, available from VWR (Radnor, PA, USA). In all cases, the cells grew at 37 °C in a tightly adjusted humified environment of 5% CO_2_–95% air.

### 2.3. Western Blot Analysis

First, 40 μg of protein was separated onto sodium dodecyl sulfate (SDS-PAGE) Tris-HCl gels. The separated proteins were transferred to nitrocellulose membranes, which were incubated for 60 min to a solution of 5% milk, to block non-specific binding sites. The membranes were exposed to the appropriate primary antibodies at a concentration of 1:1000 in a cold room for 16 h. Then, secondary antibodies (1:5000) were used to detect the corresponding primary antibodies, and a special chemiluminescent substrate was used (SuperSignal West Femto (PI34096)) to generate light, captured in ChemiDoc System from Bio-Rad (Hercules, CA, USA). VWR (Radnor, PA, USA) was the company which provided the material described in this section.

### 2.4. Fluorescein Isothiocyanate (FITC)–Dextran Assay

Paracellular permeability was estimated utilizing 24-well trans-well dishes. First, 200,000 endothelial cells were contained in each well, which were incubated for 20 min with FITC–Dextran (70 kDa, 1 mg/mL). Then, 100 mL of media was removed from each receiver well to assess fluorescence, measured with Synergy H1 Hybrid Multi-Mode Reader from Biotek (Winooski, Vermont). For this, 485 nm and 535 nm were the excitation and emission wavelengths, respectively.

### 2.5. ROS Measurement

DCFDA (25 μM) was used to measure ROS. The endothelial cells were incubated for 45 min with that compound, and the fluorescence intensity was captured by using the fluorescence plate reader described previously.

### 2.6. Densitometry and Statistical Analysis

Densitometry was carried out by using Image J software (National Institute of Health), and the data are described as Means ± SEM (standard error of the Mean). Student’s *t*-test was utilized to evaluate statistically significant differences, whereas *p* < 0.05 was considered significant. To analyze data and draw figures, we used GraphPad Prism (version 5.01). In all cases, the letter *n* is used to indicate repeats.

## 3. Results

### 3.1. GHRHAnt Protect against Toxin-Induced Barrier Dysfunction

**Figure 1.**
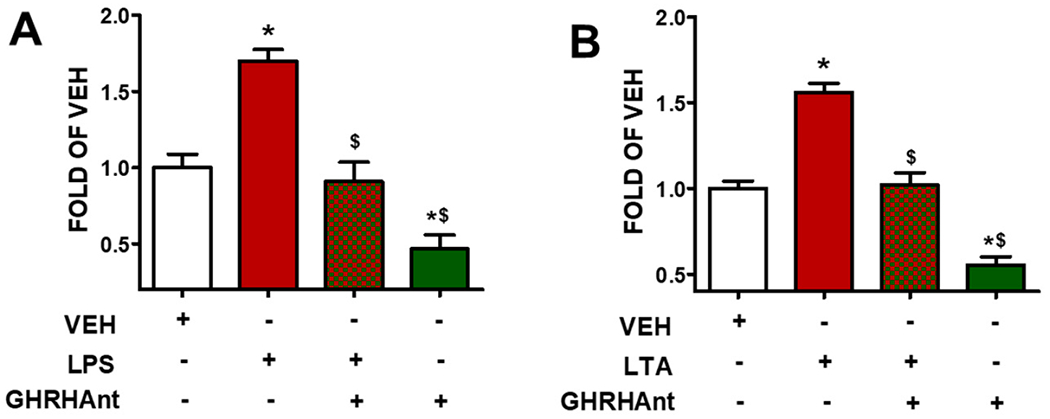
GHRHAnt protect against toxin-induced barrier dysfunction. Assessment of paracellular permeability utilizing FITC-Dextran in HUVECs exposed to vehicle (0.1% DMSO) or GHRHAnt (1 μM) for 8 h and post-treated with vehicle (PBS), or (**A**) LPS (10 μg/mL), or (**B**) LTA (10 μg/mL) (4 h). Results represent 4 independent experiments. (**A**) * *p* < 0.05 vs. VEH, ^$^ *p* < 0.05 vs. LPS. (**B**) * *p* < 0.05 vs. VEH, ^$^ *p* < 0.05 vs. LTA. *n* = 4. Means ± SEM.

Endothelial paracellular permeability was assessed to measure the barrier function. The HUVEC monolayers were treated with a vehicle (0.1% DMSO) or GHRHAnt (1 μM) for 8 h. Then, the cells were exposed to (Figure 1A) the vehicle (PBS) or LPS (10 μg/mL) and (Figure 1B) the vehicle (PBS) or LPS (10 μg/mL) for 4 h to assess their barrier function utilizing FITC–dextran (70 kDa, 1 mg/mL). GHRHAnt enhanced the endothelial barrier function and reduced the endothelial permeability.

### 3.2. GHRHAnt Counteract Toxin-Induced ROS Generation

**Figure 2.**
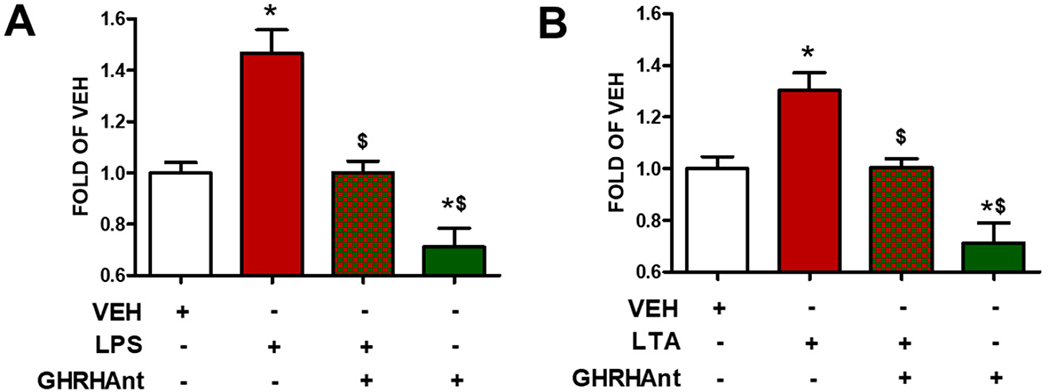
GHRHAnt counteract toxin-induced ROS generation. BPAECs exposed to vehicle (0.1% DMSO) or GHRHAnt (1 μM) for 8 h were post-treated with vehicle (PBS), (**A**) LPS (10 μg/mL), or (**B**) LTA (10 μg/mL) for 4 h. Results represent 4 independent experiments. (**A**) * *p* < 0.05 vs. VEH, ^$^ *p* < 0.05 vs. LPS. (**B**) * *p* < 0.05 vs. VEH, ^$^ *p* < 0.05 vs. LTA. *n* = 4. Means ± SEM.

BPAECs were pre-treated with GHRHAnt (1 μM) or a vehicle (0.1% DMSO) for 8 h and were subsequently exposed to (Figure 2A) LPS (10 μg/mL) or the vehicle (PBS) and (Figure 2B) LTA (10 μg/mL) or the vehicle (PBS) (4 h). Figure 2 reveals that both LPS and LTA increased the ROS levels in the BPAECs, and that this effect was counteracted by GHRHAnt. Furthermore, GHRHAnt reduced the basal endothelial ROS generation.

### 3.3. GHRHAnt Suppress Endothelial Barrier Dysfunction-Induced NEK2 Augmentation

**Figure 3.**
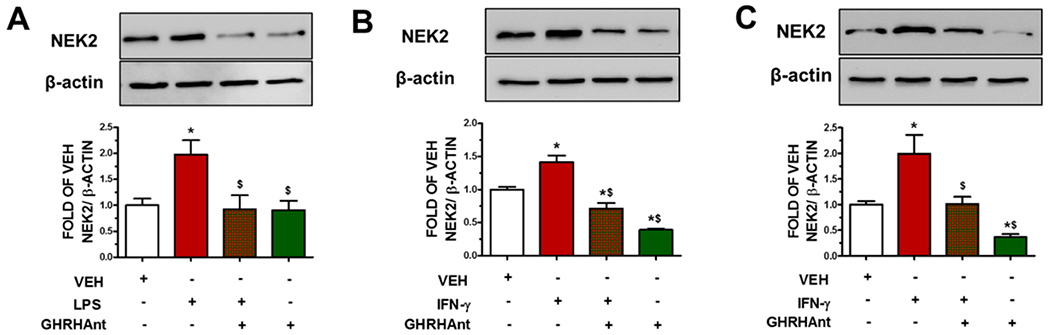
GHRHAnt suppress endothelial barrier dysfunction-induced NEK2 augmentation. (**A**) Western blot (WB) analysis of NEK2 and β-actin protein expression in BPAECs exposed to VEH (0.1% DMSO) or GHRHAnt (1 μM) (8 h) and post-treated with VEH (PBS) or LPS (10 μg/mL) (4 h). WB analysis of NEK2 and β-actin in (**B**) BPAECs and (**C**) HUVECs exposed to VEH (PBS) or IFN-γ (4 μg/mL) (24 h) and post-treated with VEH (0.1% DMSO) or GHRHAnt (1 μM) (8 h). Signal intensity was analyzed by densitometry. Protein levels of NEK2 were normalized to β-actin (loading control). Results represent 3 independent experiments. (**A**) * *p* < 0.05 vs. VEH, ^$^ *p* < 0.05 vs. LPS. (**B,C**) * *p* < 0.05 vs. VEH, ^$^ *p* < 0.05 vs. IFN-γ. *n* = 3. Means ± SEM.

Endothelial cells were exposed to GHRHAnt (1 μM) (8 h) or a vehicle and were post-treated with (Figure 3A) LPS (10 μg/mL) (4 h) or (Figure 3B,C) IFN-γ (4 μg/mL) (24 h). NEK2 was induced due to the toxin (LPS, IFN-γ) treatments. On the other hand, GHRHAnt suppressed toxin-induced barrier dysfunction.

## 4. Discussion

The endothelial cells form monolayers [22], which are tightly regulated [23]. Increased ROS generation has been associated with numerous diseases, affecting the cardiovascular and neurological systems [24]. Gram-negative bacteria can cause acute inflammatory responses by releasing inflammatory factors and ROS. LPS is the key component of Gram-negative bacteria, and it is recognized as a potent activator of monocytes/macrophages [25]. Moreover, this toxin activates cells in the immune system (e.g., macrophages, neutrophils), which further induce the synthesis of proinflammatory cytokines (e.g., IL-1β, TNF-α) [26]. LTA is a toxin of Gram-positive bacteria [27]. Figure 1 demonstrates the fact that both LPS and LTA trigger endothelial hyperpermeability. Figure 2 indicates the elevated levels of ROS generation due to LPS and LTA.

IFN-γ can potentiate the effects of LPS by upregulating the expression of CD14. This cytokine phosphorylates specific tyrosine residues on the (STAT)-1α molecule by activating cytoplasmic Janus kinases (Jak1 and Jak2) [28]. Figure 3 connects LPS and IFN–γ to NEK2 levels. LTA exerts immune-stimulating activities to induce NO production in mouse macrophages primed with IFN-γ, suggesting that it exerts a pivotal role in the pathogenesis of inflammation [29]. When the endothelial monolayer becomes leaky and inflamed, it allows for an excessive flux of innate immune cells and humoral effector molecules to cross the micro-vessel wall to the surrounding tissues. Endothelial barrier dysfunction has been associated with inflammatory lung disease and sepsis [22].

The cytokine storm resulting from inflammatory cell activation contributes to the progression of lung injury and sepsis. The former disorder has been associated with high mortality rates and causes approximately 10 million deaths worldwide each year [30]. ALI destructs pulmonary endothelial and epithelial tissues [31]. ARDS, a severe form of ALI, results from fluid accumulation in small air sacs in the lungs. This respiratory condition prevents normal lung function and results in very low oxygen levels in the blood [32].

ER assists in proteostasis, protein synthesis and folding, complex formation, transport, and destruction. Misfolded protein accumulation produces ER stress, which in turn triggers unfolded protein response activation (UPR) [33]. This complex component shields cells against a wide range of internal and extracellular threats and protects against endothelial barrier dysfunction. UPR counteracts cellular injury, and it is composed of three protein sensors, IRE1α (inositol-requiring enzyme 1α), PERK (pancreatic endoplasmic reticulum kinase), and ATF6 (activating transcription factor 6). IRE1 in its inactive state binds with the UPR marker binding immunoglobulin protein (BiP). IRE1 self-associates during increased ER stress conditions, and it was suggested that IRE1 and improperly folded proteins can interact directly [34]. ATF6 can sense ER stress, and it is then translocated to the nucleus to promote gene expression, which corresponds to ER chaperones and other protein-folding enzymes [35]. ATF6 protects against barrier dysfunction. PERK is involved in cytotoxicity, reduces SOX2, and controls filamentous actin via Filamin-A. That function is involved in the adaptive response to mechanical stress and in hyperpermeability responses [36]. UPR activation boosts anti-inflammatory responses and protects against barrier dysfunction. GHRHAnt can activate UPR.

GHRH is produced by the hypothalamus and triggers growth hormone release by the somatotropic cells of the anterior pituitary gland [37]. Elevated GHRH secretion due to physical and mental stress is blocked by a hypothalamic neurohormone called somatostatin. The secretion of this hypothalamic peptide is also inhibited by insulin-like growth factors via mechanisms based on negative feedback inhibition. GHRHAnt oppose the growth factor activities of GHRH in malignancies and suppress cancers by blocking GHRHR/SVs activation. The anticancer properties of these analogues are partially attributed to insulinlike growth factor I (IGF-I) suppression. It has been suggested that there is a positive relationship between the plasma IGF-I levels and the chance of developing colorectal, breast, and prostate cancer. GHRHAnt oppose endothelial barrier dysfunction, induce the barrier enhancer P53, and activate UPR to support barrier integrity [4]. Pathways that are involved in the mediation of GHRH-related analogs in endothelial inflammation have been discussed previously [10].

NEK2 is essential for breast carcinoma carcinogenesis, tumor invasion, and tumorigenic growth. It is attached to beta-catenin and phosphorylate p53 at Ser315, which in turn destabilizes P53. NEK2 can function in the cytoplasm as well as in the nucleus via localization modulation, due to alternative splicing. Moreover, it has recently been implicated in inflammatory processes [14,21].

## 5. Conclusions

The present study demonstrates the protective activities of GHRHAnt in endothelial cells exposed to bacterial toxins and involve NEK2 in such events, suggesting a possible multifaceted role of that kinase in disease pathogenesis, including inflammatory lung disorders. Based on our observations, NEK2 has the potential to serve as a therapeutic target in diseases relating to endothelial activation and leak, and the development of targeted NEK2 inhibitors may appear to be an intriguing strategy to treat abnormalities related to NEK2. However, more studies are needed to further substantiate our speculations, since the experiments of the present study were conducted exclusively in endothelial cells, in vitro. Future approaches in mice who are exposed to GHRHAnt and bacterial toxins, as well as in genetically modified rodents which do not express NEK2, will strengthen our observations.

## Data Availability

Data are available upon reasonable request.

## Abbreviations

ECs: endothelial cells
NADPH: Nicotinamide adenine dinucleotide phosphate
eNOS: Endothelial Nitric Oxide Synthase
IL-1β: Interleukin 1 beta
LPS: Lipopolysaccharides
TNF-α: Tumor necrosis factor-alpha
LTA: Lipoteichoic acid
IFN-γ: Interferon gamma
CD14: Cluster of differentiation 14
NO: Nitric oxide
ALI: Acute lung injury
ARDS: acute respiratory distress syndrome
ER: Endoplasmic Reticulum
SOX2: Sex determining region Y-box 2
NEK2: never in mitosis A (NIMA)-related kinase 2
GHRH: growth hormone-releasing hormone
GHRHAnts: growth hormone-releasing hormone antagonists
EBD: endothelial barrier dysfunction
GHRH-R: growth hormone–releasing hormone receptor
SVs: splice variants
